# Epithelial-to-Mesenchymal Transition in Metastasis: Focus on Laryngeal Carcinoma

**DOI:** 10.3390/biomedicines10092148

**Published:** 2022-09-01

**Authors:** Anastasios Goulioumis, Kostis Gyftopoulos

**Affiliations:** 1 Department of Anatomy, University of Patras School of Medicine, 26504 Patras, Greece; 2 ENT Department, “Karamandanio” Pediatric Hospital of Patras, 26331 Patras, Greece

**Keywords:** epithelial to mesenchymal transition (EMT), laryngeal carcinoma, metastasis

## Abstract

In epithelial neoplasms, such as laryngeal carcinoma, the survival indexes deteriorate abruptly when the tumor becomes metastatic. A molecular phenomenon that normally appears during embryogenesis, epithelial-to-mesenchymal transition (EMT), is reactivated at the initial stage of metastasis when tumor cells invade the adjacent stroma. The hallmarks of this phenomenon are the abolishment of the epithelial and acquisition of mesenchymal traits by tumor cells which enhance their migratory capacity. EMT signaling is mediated by complex molecular pathways that regulate the expression of crucial molecules contributing to the tumor’s metastatic potential. Effectors of EMT include loss of adhesion, cytoskeleton remodeling, evasion of apoptosis and immune surveillance, upregulation of metalloproteinases, neovascularization, acquisition of stem-cell properties, and the activation of tumor stroma. However, the current approach to EMT involves a holistic model that incorporates the acquisition of potentials beyond mesenchymal transition. As EMT is inevitably associated with a reverse mesenchymal-to-epithelial transition (MET), a model of partial EMT is currently accepted, signifying the cell plasticity associated with invasion and metastasis. In this review, we identify the cumulative evidence which suggests that various aspects of EMT theory apply to laryngeal carcinoma, a tumor of significant morbidity and mortality, introducing novel molecular targets with prognostic and therapeutic potential.

## 1. Introduction

Laryngeal carcinoma accounts approximately for 1% of all malignant diseases diagnosed annually worldwide. Nevertheless, its incidence has increased by 12% during the last three decades [1]. It is a tumor of significant morbidity because it affects a multi-role organ that functions as an airway and serves in communication, swallowing, and respiratory system protection. Despite the evolving surgical and medical approaches, 20 to 50% of patients die due to the progression of this tumor [1].

In epithelial tumors, such as laryngeal carcinomas, a notable morbidity and mortality deterioration occurs when they become metastatic. Metastasis is a multistep process that commences with the invasion of tumor cells in their stroma and proceeds with the intravasation of cancer cells into blood and lymph vessels. Subsequently, malignant cells remain dormant until they find favorable microenvironment conditions in distant tissues. At that stage, the homing of malignant cells occurs, and eventually, a new metastatic niche develops [2].

Basic-research results are translated into various targeted molecular therapies that enrich the armamentarium of clinicians. These therapies may target different stages of the metastatic process. However, since a carcinoma that has metastasized is considered already a systematic disease, the earlier stage we target, the more effective our therapeutic approaches can be. Thus, elucidating the molecular phenomena that endow epithelial tumor cells with the migratory capacity to leave their “neighborhood” can significantly contribute to the battle against carcinoma metastasis. Several studies indicate that during the initial step, where invasion in the stroma occurs, carcinoma cells abolish their epithelial and acquire mesenchymal traits. These alterations constitute a molecular phenomenon, studied for more than three decades in cancer metastasis, called epithelial-to-mesenchymal transitions (EMT) [3,4].

## 2. Epithelial-to-Mesenchymal Transition (EMT)

**Figure 1:**
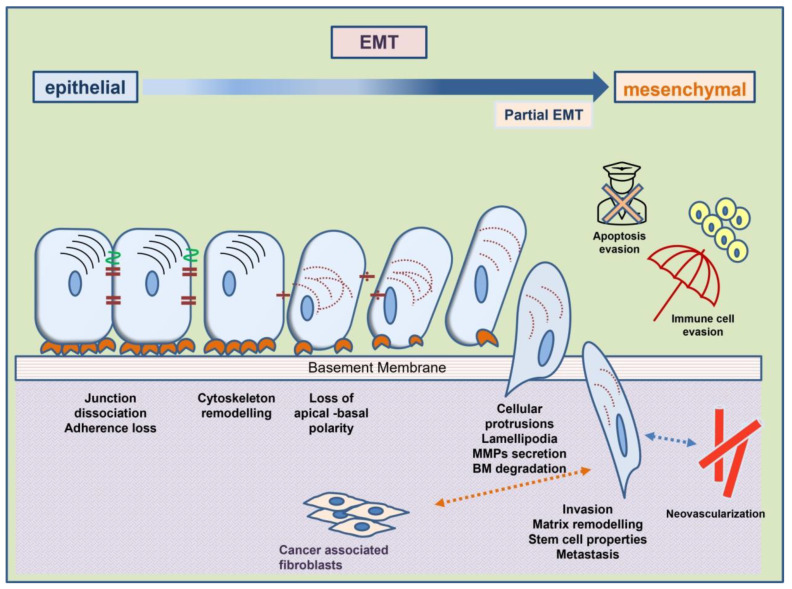
Cellular phenotypic changes and acquired mesenchymal characteristics in the course of mesenchymal-to-epithelial transition provide tumor cells with increased metastatic properties. Interaction with the tumor microenvironment is an important step.

Epithelial-to-mesenchymal transition is a well-studied phenomenon in embryology and occurs during the morphogenesis of organs [3]. This case is described as EMT type I. The molecular procedure of EMT is also reprogrammed in the healing of wounds and the pathological fibrosis of organs, known as EMT type II [5]. EMT III is the type that is implicated in tumor metastasis [6]. While initially focusing on the abolishment of epithelial and acquisition of mesenchymal characteristics by the tumor cells, the idea behind EMT currently incorporates all the phenotypic and molecular characteristics that enable tumor cells to migrate, survive, and proliferate in distant tissues. In other words, it is a complete model of molecular processes signaled by specific factors called inducers. This model progresses via cross-linked molecular pathways, concluding with functional and structural modifications that make the carcinoma cells metastatic. These modifications are mediated by molecules known as the effectors of EMT (Figure 1).

### 2.1. The Role of EMT Effectors in EMT Characteristics

#### 2.1.1. Loss of Adhesion

One of the fundamental characteristics of epithelial cells on the mucosal surfaces is their polarity. An additional characteristic is the existence of adhesion structures that attach every epithelial cell with its adjacent cell and the basal membrane. Such adhesion structures, including tight junctions, zona occludens, desmosomes, and hemidesmosomes, are constructed by adhesion molecules, such as E-cadherins [7]. E-cadherins are transmembrane proteins that create a homophilic bond with E-cadherins of the adjacent cell. At the same time, E-cadherins are anchored to the actin filaments of the cytoskeleton via a molecular complex consisting of p-120 and β-catenins. A hallmark of EMT is the abolishment of E-cadherins. The loss of E-cadherin can happen mainly when its promoter is downregulated by transcription factors, especially Snail, Slug, and ZEB2 [8]. Secondarily, E-cadherins can be abolished due to their cleavage by metalloproteinases (MMPs) or their phosphorylation and subsequent degradation by the ubiquitin–proteasome system [9,10]. E-cadherin abolishment results in the epithelial cells’ detachment from each other and the basement membrane. Due to the dismantling of the molecular complex, p-120 and β-catenins are released into the cytoplasm. When these catenins reach the nucleus, they function as transcription factors that enhance the expression of crucial molecules, such as MMPs and N-cadherin that drive EMT progression. The “cadherin switch” is a critical event that enhances tumor cells’ migratory capacity [11].

In laryngeal carcinoma clinical samples studied with immunohistochemistry, advanced staging and decreased differentiation correlate with E-cadherin downregulation, β-catenin nuclear translocation, and expression of the transcription factors Snail, Slug, and ZEB2 [12,13]. Co-expression of E-cadherin/β-catenin in laryngeal tissue samples also correlates with clinicopathological parameters such as lymph node metastasis and overall patient survival [13,14]. Additionally, a cadherin switch, with N-cadherin replacing E-cadherin, is evident in laryngeal carcinoma cell lines [13,15]. The Twist transcription factor regulates N-cadherin expression in laryngeal carcinoma cell lines [15]. Another study on Hep-2 cell lines has shown that the knockdown of Snail can inhibit EMT [16].

#### 2.1.2. Remodeling of the Cytoskeleton

During the course of EMT, epithelial cells undergo alterations of their cytoskeleton. Activation of Rho GTPases by free p-120 catenins leads to another hallmark trait of EMT, the substitution of normal epithelial cytokeratins by vimentin [17,18,19,20]. Modifications of the cytoskeleton, additionally, contribute to the loss of polarity, the acquisition of spindle cell morphology, and the creation of structures that facilitate migration, such as lamellipodia [21,22].

When EMT develops, typical modifications in the cytoskeleton occur in laryngeal carcinoma tissue samples, with the replacement of cytokeratins by vimentin in higher-grade neoplasms [23]. Spindle-cell squamous laryngeal carcinoma is a rare entity whose nature is not clarified. However, it is shown in other carcinomas that spindle-cell morphology is acquired during EMT [24]. Interestingly, one study supports that laryngeal carcinoma cells that metastasize through lymphatic vessels are exposed to fluid shear stress that can induce EMT, cytoskeleton modifications, and lamellipodia formation [25]. Lamellipodia enhance local invasiveness in different head and neck carcinomas [25,26].

#### 2.1.3. Evasion of Apoptosis

Apoptosis is a programmed cell death met both in pathological and physiological situations in tissues. Apoptosis is mediated by molecules known as caspases, which can be activated by various molecular pathways [27]. An apoptotic cell is removed without leaving behind an inflammatory reaction. One typical condition that induces a type of apoptosis in epithelial cells, known as anoikis, is their detachment from the basal membrane [28,29]. The basal membrane sends survival signals to the cells through the integrins of the epithelial cells’ focal adhesions [30]. Normally, the epithelial tumor cells that detach from the basal membrane while becoming metastatic should die by anoikis. However, through EMT, molecular pathways such as ILK-Akt and MAPKs preserve the tumor cell’s survival signals [31,32]. Notably, apoptosis evasion is related to resistance to various chemotherapeutic drugs [33].

On the other hand, specific molecular pathways activated during EMT lead tumor cells to a condition called autophagy, a catabolic process that normally aids cellular homeostasis by eliminating damaged organelles and molecules. In cancer-associated autophagy, the tumor cells intentionally destroy some of their organelles to reduce their energy needs. This way, tumor cells can remain dormant until they find favorable conditions to proliferate again [34]. The interplay between EMT and autophagy is complex: autophagy may initiate or suppress EMT but is also activated by EMT-related signaling pathways, i.e., hypoxia or TGF-b [34]. One of the most important regulators of autophagy, mTOR, is also a downstream effector of the PI3K-Akt pathway of EMT [35]. However, the final effect of EMT on autophagy may be related to the cell type and the stage of tumor progression: in early phases of tumorigenesis, autophagy may impede the EMT process; on the other hand, once cells have undergone EMT, they may promote and utilize autophagy as a means of apoptosis and immune surveillance evasion, increasing their metastatic potential [36].

In several head and neck neoplasias, including laryngeal carcinoma, activation of focal adhesion kinase (FAK) has been shown to protect cancer cells from caspase-mediated anoikis [37]. Caspases have been suggested as promising targets for laryngeal carcinoma therapy [38]. Additionally, studies with cell cultures have shown retinoids to suppress FAK, leading to apoptosis and G2/M cell cycle blockage [39].

Novel molecules involved in apoptosis, such as the tumor suppressor programmed cell death 4 protein (PCDP4), have shown emerging roles in EMT of the laryngeal carcinoma contributing to the cadherin switch process. In vivo experiments in mice have shown that PCD4 silencing is associated with dysregulation of the Wnt-β-catenin and the STAT3-miR-21 signaling pathways, suggesting crosstalk of PDCD4 with important regulators of EMT [40].

#### 2.1.4. Evasion of Immune Surveillance

Transcription factors activated during EMT lead to the expression of cytokines, such as TGF-β, which also functions as a master immune regulator [41,42]. As evident in several neoplasias, enhanced expression of TGF-β may prevent the recognition of tumor cells by T-helper lymphocytes [43]. Additionally, EMT leads cancer cells to reduce the expression of HLA proteins of the histocompatibility system and eventually to evasion of immune surveillance [44].

In laryngeal carcinoma, TGF-β regulates cells of the innate and adaptive immune system [45]. Upon exposure to TGF-β, the primary antigen-presenting cells, the dendritic cells, facilitate immune tolerance and become inactive and immobile [46]. Moreover, TGF-β skews T-naïve lymphocytes’ differentiation towards T-regulatory lymphocytes (T-regs) instead of the immune-potent Th1 lymphocytes [45]. This condition renders the immune surveillance of malignant cells in laryngeal carcinoma less effective.

#### 2.1.5. Upregulation of Metalloproteinases (MMPs)

Transcription factors such as AP-1 and β-catenin, which accumulate in the nucleus as a consequence of the activation of the molecular pathways of EMT, upregulate the expression of the metalloproteinases [47]. MMPs enhance tumor cells’ migratory capacity in several ways. They can activate signaling molecules for EMT, such asFGF, and cleave E-cadherin, which leads to dismantling adhesion structures [48,49]. They also participate in cytoskeleton remodeling and the evasion of apoptosis [50,51]. Additionally, MMPs can activate VEGF, a crucial factor for neovascularization [52]. Moreover, MMPs on the lamellipodia can pave the way through stroma by degradation of collagen fibers [53]. Finally, substances that emerge from stroma after cleavage by MMPs, such as Elastin, can act as chemotactic factors that contribute to the migratory capacity of tumor cells [54].

Less differentiated laryngeal carcinomas appear with enhanced expression of metalloproteinases such as MMP-2 and MMP-9 and suppressed expression of the tissue inhibitors of metalloproteinases TIMP -1 and -2 [55]. Thus, metalloproteinase expression has been suggested as a prognostic factor for laryngeal carcinoma [56]. Other immunohistochemical studies have additionally shown that the primary source of MMP-9 in laryngeal carcinoma that promotes EMT is the inflammatory cells in the tumor stroma [57].

#### 2.1.6. Neovascularization

The formation of new vessels (neovascularization or neoangiogenesis) is a crucial procedure for developing metastasis since it facilitates the supply of oxygen and nutrients to solid tumor cells for a tumor to grow beyond a certain size (1–2 mm^3^ in diameter) [58]. These newly formed vessels are usually small in diameter; hence, the measurement of microvessel density (MVD) is a long-established method to quantify associated neoangiogenesis in several tumors [59]. Two of the molecular pathways that function as master regulators of EMT, RTKs—Ras—MAPK and Src—PI3K—Akt, contribute to neovascularization [60]. Additionally, the upregulation of MMPs is related to the activation of VEGF-A, a growth factor implicated in creating new blood vessels [61]. MMPs also contribute to VEGF-D activation, which is implicated in forming new lymph vessels [62]. Interestingly, enhanced expression of the EMT-related transcription factor Twist is involved in the phenomenon of vascular mimicry [63]. This phenomenon consists of channels in the cancer mass that imitate vessels, which are not typically formed by endothelial cells. Nevertheless, this type of pseudovessels can also pave the way for metastasis of the epithelial tumor cells [64].

Available data suggest that in laryngeal carcinoma tissue samples, molecular pathways that intersect and end up with the activation of the transcription factor ZEB2 mediate the mesenchymal transition of the epithelial cells and upregulate the expression of genes that mediate neovascular formation by activated endothelial cells [65]. Moreover, vascular mimicry has also been studied in laryngeal carcinomas, showing enhancement of lymph node metastasis [66]. Vascular mimicry has been correlated with clinicopathological parameters in laryngeal carcinomas, such as histopathology grade and disease-specific and metastasis-free survival [66]. Finally, lymphangiogenesis in the early stages also contributes to the dissemination of laryngeal carcinoma and is related to local and locoregional recurrence [67].

#### 2.1.7. Acquisition of Stem-Cell Traits

A proportion of cells in every tissue appear with stem cell properties, the most characteristic being self-renewal and potency [68]. Self-renewal is the ability of the cells to go through numerous cycles of division while maintaining their undifferentiated state. Potency is the capacity to differentiate into specialized cell types. These properties are crucial for metastasis but also for resistance to chemotherapeutics. Cells acquire characteristic surface antigens of stemness, typically CD44+, under the regulation of molecular pathways of EMT, such as the pathways of TGF-β, Wnt/β-catenin, Notch/Hedgehog, and miRNAs [6]. A possible model that has been suggested is that among the tumor cells, those which will undergo EMT acquire stem-cell properties and promote metastasis.

In the laryngeal carcinoma cell line, Hep-2, the downregulation of miRNA-145 contributed to the acquisition of stem-cell properties, such as potency and self-renewal, by carcinoma cells, such as the expression of CD-133 [69]. This condition occurs mainly in hypoxic environments and is related to higher proliferation and colony formation ability of the Hep-2 cell lines [70]. Additional markers associated with stemness in laryngeal carcinoma include the expression of aldehyde dehydrogenase (ALDH) and the cell-surface glycoprotein CD44+ [70]. More specifically, CD44+ functions as a receptor for hyaluronic acid involved in cell adhesion and migration. Carcinoma cell lines that acquired stem-cell properties exhibit self-regeneration and resistance to radiotherapy [71]. Eventually, these conditions are related to poorer clinicopathological parameters, such as advanced stage and recurrence in laryngeal carcinoma [72].

#### 2.1.8. Altered Interaction of EMT Cells with Tumor Stroma

Tumor cells develop a particular interaction with the underlying stroma that promotes migration and metastasis. Tumor cells exert functional modifications to the tissue stroma leading to its activation. Subsequently, the activated stroma cells mediate the EMT of the tumor cells by contributing to the signaling of this molecular phenomenon [73,74]. A new cell, the cancer-associated fibroblast (CAF), appears in the activated stroma, characterized by the presence of alpha-smooth muscle actin (α-SMA) [75]. Several theories speculate on the origin of CAFs. The most prevalent theories support their origin from preexisting fibroblasts transformed under the effect of TGF-β and IL-6 produced by the tumor and inflammation cells [76]. In a vicious circle, tumor cells create CAFs, and, subsequently, CAFs induce EMT of tumor cells through Wnt, FGF, and TGF-β production, endowing them with a migratory capacity [74,77]. Additionally, CAFs produce MMPs, angiogenetic, and chemotactic factors for inflammatory cells, mediating multiple stages of the molecular process of metastasis. It is noteworthy that interleukins, miRNAs, and growth factors from the tumor site can travel in exosomes and activate the stroma of distant tissues [75]. Exosomes are nanometer-scale membrane vesicles deriving from the tumor and tumor-microenvironment cells and are released into the circulation. Exosomes can prepare the microenvironment of the future metastatic niche, demonstrating a refined manifestation of the “seed and soil” theory [78].

In the larynx, α-SMA-expressing CAFs in the stroma are detected only in invasive carcinoma and not in the stroma of the adjacent normal tissue [79,80]. Moreover, several studies have shown that miRNAs that have selectively enriched exosomes, such as miR 1246, miR 1290, miR 335 5p, miR 127 3p, and miR 122 5p, are distinct compared to miRNAs in the parental carcinoma cells [81]. Crucial exosomalmiRNAs derived from the CAFs of laryngeal carcinoma stroma promote migration and invasion, as shown in cultures from cancer specimens [82,83].

### 2.2. Inducers and Pathways of EMT

EMT is initiated by signaling molecules and develops through the activation of complicated and cross-linked pathways that usually conclude to transcription factors in the nucleus that regulate the expression of genes with critical roles in EMT.

There are multiple inducers for EMT derived mainly from CAFs and inflammatory cells. The most extensively studied signaling molecules are TGF-β, Wnt, growth factors such as HGF, EFG, FGF, and stromal components signaling through integrins [84].

#### 2.2.1. The Pathway of TGF-β

TGF-β is secreted by epithelial tumor cells and CAFs of the activated stroma [85]. It actuates its molecular pathway through transmembrane receptors that activate a family of cytoplasmic molecules called Smads [85]. Smads form complexes that function as transcription factors in the nucleus to express or repress crucial molecules for EMT. Interestingly, the TGF-β pathway may present both tumor-promoting and tumor-suppressive roles [86]. This dual role is related to the co-activators or co-repressors of the Smad complexes in the nucleus, the type of promoter of the various genes, and the different molecular pathways that are activated in the specific cell at a given time [86]. Moreover, the TGF-β signaling, apart from canonical, is also triggering non-canonical pathways. Typical molecular pathways activated during EMT, such as Wnt, Ras, and PI3K-Ras, which are cross-linked with TGF-β canonical pathway, can affect the final result of TGF-β signaling [87].

There are several indications of the dual role of TGF-β in laryngeal carcinoma. TGF-β has an enhanced expression in laryngeal carcinoma compared to the adjacent normal tissues, supporting its tumor-promoting role [88]. Additionally, TGF-β pathway downstream genes act as an immune suppressor in head and neck carcinomas, negatively affecting the survival indexes [89]. In another study, the downregulation of the TGF-β receptor II is shown to be an early event in carcinogenesis, indicating the TGF-β pathway tumor-suppressive role at the initial stages of carcinoma progression [90].

#### 2.2.2. Molecular Pathway of Wnt

The typical hallmark of EMT is the downregulation of the adhesion molecule E-cadherin. A consequence of this condition is the release in the cytoplasm of β-catenins. B-catenins form a complex with E-cadherins, and when they are set free in the cytoplasm, they normally bind on a molecular complex formed by APC, Gsk-3b, and Axin. This complex leads β-catenin to degradation by the ubiquitin-proteasome system [91]. Cancer-associated fibroblasts in the activated stroma of tumor cells secrete Wnt molecules [92]. Wnt induces EMT, signaling via a Frizzled (Fz) family transmembrane receptor. When the receptor is activated, a signal is transmitted to the phosphoprotein Dishevelled (Dsh), which leads to translocation of the negative Wnt regulator, Axin. The stereotactic alteration of the complex reduces the affinity of β-catenins to it, thus, leading to the accumulation of β-catenin in the cytoplasm and subsequently translocation in the nucleus, where β-catenin functions as a transcription co-factor of TCF/LEF. This complex of transcription factors is crucial for the molecular modifications of EMT on the tumor cells [84]. Recently, non-canonical Wnt pathways have been described to cross-link other EMT molecular pathways [93].

In laryngeal carcinoma, several long coding RNAs have been shown to signal via the Wnt/β-catenin pathway [94,95]. Other long coding RNAs, such as NEF, exhibit a tumor-suppressive role targeting the Wnt pathway [96]. Additionally, in laryngeal carcinoma tissue samples and cell lines, the FOXP4 transcription factor regulates EMT as it directly bounds to the LEF promoter, a downstream effector of the Wnt pathway [97]. Moreover, even the main effector of the Hippo signaling pathway, Yes-associated protein, is implicated in the Wnt/β-catenin pathway in laryngeal carcinoma cell lines [98]. Finally, it has been shown that aberrant Wnt signaling has a prognostic value in early laryngeal carcinomas [99].

#### 2.2.3. Molecular Pathways of Growth Factors

Growth factors reach tumor cells via paracrine and autocrine loops. Their signaling is mediated through the cell-surface receptors of tyrosine kinase (RTKs) [100]. The molecular pathways that RTKs activate include Ras [101], Rho [102], Src [103], and Notch-Hedgehog [104], which participate in the majority of the molecular phenomena that define EMT.

The Src molecule plays a crucial role in laryngeal carcinoma pathogenesis since siRNA silences inhibit carcinoma growth and regulates apoptosis through the Src/PI3K/Akt pathway in vitro and in vivo [105,106]. Notch 1 and 2 are other molecules implicated in cell growth, aberrant angiogenesis, acquisition of stem cell traits, anti-apoptosis, and the metastasis of laryngeal carcinoma [107,108]. Knockdown of Notch 1 in Hep-2 cells inhibited molecules with nodal roles in the development of EMT, such as p-Akt, cyclin D1, and Bcl-2 [109]. Moreover, K-Ras over-expression has been correlated with dedifferentiation in laryngeal carcinoma cells [110]. Ras has also been found to be regulated by miR-21 in the larynx. More specifically, inhibition of miR-21 by antisense oligonucleotides led to decreased levels of Ras and significant suppression of laryngeal tumor cells’ migratory capacity [111].

#### 2.2.4. Signaling of EMT via Integrins

Integrins participate in forming transmembrane complexes known as focal adhesions [112,113,114]. Focal adhesions function as adhesion molecules between epithelial cells and the basal membrane and signal transduction units between the cell and its stroma. The molecular pathways activated by Integrins participate in the evasion of apoptosis, migration, and proliferation of tumor cells [115].

The expression of the integrin superfamily in laryngeal carcinoma has been correlated with angiogenesis and lymphatic metastasis [116]. Integrin β1 enhanced inherent radio-resistance in Hep-2 cell lines [117]. Several studies have implicated ανβ5 expression in EMT and metastasis in laryngeal carcinoma. Available data suggest inhibiting Integrin-subunit expression may hinder Hep-2 cell proliferation and lead to apoptosis [118,119,120].

#### 2.2.5. Epigenetic Regulation of EMT by microRNAs

**Figure 2:**
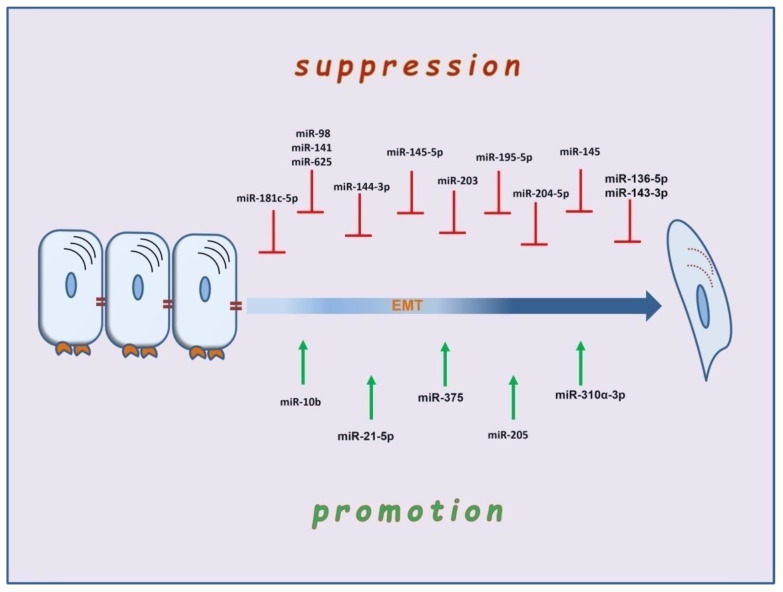
The multifaceted role of microRNAs in suppression and promotion of EMT in laryngeal carcinoma.

MicroRNAs (miRNAs) are 22-nucleotide, non-coding RNAs that function in mRNA silencing, thus regulating gene expression at a post-transcriptional level [121,122,123]. One of the most widely studied families is the miR-200, which typically suppresses the expression of ZEB1 and 2, comprises two transcription factors involved in suppressing the E-boxes of the E-cadherin gene [124]. During the EMT course, the enhanced expression of TGF-β downregulates the expression of miR-200, thus indirectly leading to the abolishment of adhesion [125]. Different miRNAs, such as microRNA-10b, are upregulated by the EMT transcription factor Τwist [126]. The upregulation of miRNAs can lead to enhanced expression of Rho GTPases related to cytoskeleton remodeling, a nodal event for EMT development [127]. Apart from miRNAs, several epigenetic modifications that mediate EMT with methylation and demethylation of the promoters of genes, such as the gene that encodes E-cadherin, have been described [128]. There is a developing literature on the role of miRNAs in the pathogenesis of laryngeal carcinoma metastasis [129] (Figure 2). In most cases, miRNAs act as suppressors of proliferation and lymph-node metastasis and inducers of apoptosis, as in the cases of miR-141, miR-136-5p, miR-204-5p, miR-143-3p, miR-145-5p, and miR-144-3p [130,131,132,133,134,135]. Like miR-98, miR-625, and miR-195-5p, other microRNAs can reverse EMT [136,137,138,139]. Downregulation of miR-203 leads the laryngeal carcinoma cells to acquire stem-cell traits, such as CD44+ [139]. However, other miRNAs, such as miR-10b, act as a promoter of EMT in Hep-2 cells by enhancing the expression of N-cadherin and decreasing the expression of E-cadherin. The cadherin switch, in this case, develops under the effect of molecular mechanisms distinct from the typical EMT transcription factor-mediated regulation of genes. Thus, in miR-10b-mediated E-cadherin downregulation, the mRNA of Snail, ZEB, and Twist remains unchanged [140]. Accordingly, miRNA-205 and miR-375 regulate the invasion of laryngeal carcinoma cells via Akt-mediated EMT [141]. Available studies also indicate the tumor-promoting roles of miR-21-5p and miR-310a-3p through KLF6 expression and regulation of Smad4, respectively [142,143].

#### 2.2.6. Regulation of EMT by Inflammation and Hypoxia

Inflammation is associated with the induction and promotion of tumorigenesis in several tumors. The Wnt and TGF-b signals produced by macrophages can induce EMT of tumor cells through inflammatory reaction [144]. Interestingly, cyclooxygenase regulates the Smad family of molecules, switching the TGF-β pathways function from tumor-suppressive to tumor-promoting [145].

In laryngeal carcinoma, gastroesophageal reflux and the subsequent inflammatory condition induced are associated with carcinogenesis. More specifically, pepsin promotes IL-8 signaling-induced EMT that can be reversed with the inhibition of pepsin [146]. IL-8 has been shown to regulate several transcription factors driving EMT, such as Smads, NF-κB, STAT3, Snail, Twist, and Zeb-2 [147].

Hypoxia induces the expression of transcription factors, such as the Hypoxia-inducible factor (HIF-1α), that respond to a decrease in available oxygen in the cellular environment [148]. The molecular pathways downstream-effectors of HIF-1α cross-link with the typical EMT pathways that conclude to the expression of Snail or Twist [149].

In laryngeal carcinoma, it has been shown that hypoxia inhibits the oxidative phosphorylation process and enhances aerobic glycolysis [150]. Aerobic glycolysis is a molecular process where glucose transporters (GLUTs) are implicated. Glucose transporters may contribute to cancer development by activating the NF-kB and PI3K/Akt pathways [151]. These are typical molecular pathways that are activated during the EMT in the course of metastasis. Moreover, HIF-1α may upregulate GLUT-1 expression in laryngeal carcinoma leading to chemo-resistance. Chemo-resistance is shown in vitro to be reversed by inhibition of GLUT-1 [152]. Other studies have related HIF-1α with worse clinicopathological parameters, such as the clinical stage, histological differentiation, and lymph node metastasis [153]. Additionally, in laryngeal carcinoma cell cultures, HIF-1α upregulates the expression of Survivin, a member of an inhibitor-apoptosis protein family [154].

## 3. Epithelial-to-Mesenchymal Transition—The Contemporary Approach

Epithelial-to-mesenchymal transition is a molecular phenomenon widely studied in embryogenesis and highly tenable in the metastasis of carcinomas. Interestingly, medical imaging methods have shown the migration of epithelial tumor cells in mammary carcinoma [155]. Epithelial-to-mesenchymal transition has been studied in synthetic 3D polymers that can imitate the epithelium–endothelium microenvironment and invivo with genetically modified mice [156].

However, some facts render the EMT theory in cancer metastasis debatable. EMT is neither stable nor discernable in any stage of metastasis. It is widely accepted that tumor cells dedifferentiate and are characterized by aberrant expression of molecules, not necessarily related to EMT. Additionally, the pleomorphism of epithelial cells can probably, explain the spindle morphology of epithelial tumor cells in some cases. Finally, many studies have shown that epithelial carcinomas metastasize, preserving their epithelial traits, such as adhesion molecules [157]. It is noteworthy that mesenchymal characteristics do not seem beneficial for tumor cells in every stage of metastasis. A tumor cell requires its epithelial properties to perform homing in distant tissues. At this stage, a reverse condition, mesenchymal-to-epithelial transition (MET), seems to occur [158]. This phenomenon is already described in the morphogenesis of kidneys [159]. In cancer metastasis, MET is the final event that allows cancer cells to re-acquire their epithelial characteristics and establish themselves at the site of metastasis, also known as the metastatic niche.

Currently, the approach to EMT is redefined to incorporate the new evidence. Epithelial-to-mesenchymal transition is not an all-or-none binary phenomenon. Cumulative data support that it is more probable to detect hybrid cells in the migrating tumor with properties of both epithelial and mesenchymal cells, a phenomenon described as partial EMT (p-EMT) [160]. The balance of a tumor cell between the two states can be attributed to the expression levels of transcription factors, such as Snail, or to the level of miRNAs at a given time [161]. Different patterns of metabolic plasticity may also have a role in the Epithelial–Hybrid–Mesenchymal spectrum in tumors [162]. Notably, only such hybrid cells can express CD44+ surface-glycoproteins that characterize stem cells [163]. The concept of p-EMT and its potential metastatic benefits has also been described in studies on head and neck carcinomas [164,165,166,167]. More importantly, p-EMT has been shown to be related to detrimental clinical effects: a higher p-EMT score was found to be related to lymph node metastasis and is associated with advanced lymph node staging in head and neck squamous cell carcinoma (HNSCC) [168]. Similarly, p-EMT is directly related to a higher metastatic potential in FAT1-mutated human squamous cell carcinomas [169].

Another interesting observation is that tumor cells may not migrate individually but as cell complexes [170]. This phenomenon has also been described as collective migration, where clusters of cells rather than single cells drive the metastatic process [171]. There is no need for all tumor cells to undergo a complete transition in such a case. This assumption may explain why some tumor cells migrate, preserving their epithelial character. Interestingly, p-EMT seems to play a crucial role in the collective migration process, as these hybrid cells preserve their plasticity, evade apoptosis, and resist immune surveillance more effectively [168]. Fibroblasts and platelets may also participate in these mixed cell complexes formed by tumor cells. The former secrete crucial signaling molecules for EMT, such as TGF-β and growth Factors [172]. It is also noteworthy that the state of p-EMT also affects the motility dynamics of the cell cluster morphology; the p-EMT state of hybrid cells seems to enable cell clusters to metastasize in spheres, retaining some degree of cell adhesion, while full EMT cells convert to a more spindle-such as morphology and migrate as single cells [171,173]. The EMT state of different tumor cells also seems to defer according to their localization at the initial tumor site: cells at the invasive front of the tumor have more mesenchymal characteristics and pave the way through the ECM (leader cells) compared to cells at the back of the cluster that retain more epithelial traits (follower cells) [171,174].

**Figure 3:**
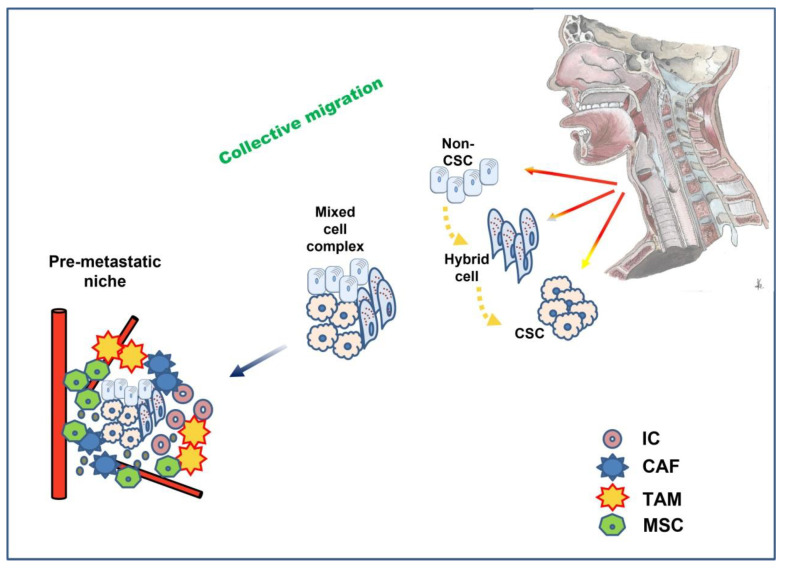
Partial (hybrid) EMT plays a key role in collective migration: clusters of mixed cancer cells in different stages of EMT and stemness invade and travel through the circulation to the premetastatic niche, where interaction with a variety of cells promotes MET and eventually successful seeding at the distant site. CSC: cancer stem cell; IC: immune cell; TAM: tumor-associated macrophages; CAF: cancer-associated fibroblast; MSC: mesenchymal stem cell.

In line with previous approaches to the EMT phenomenon, detecting the specific cells that promote metastasis in the tumor mass was focused on the invasive front of the migrating tumor [175]. However, this may not be the case. Currently, chasing metastatic cells, the investigation focuses on a small group that reaches 5% of the tumor mass, the stem cells. Although molecular pathways of EMT can lead tumor cells to acquire stem cell properties, plasticity, per se, is not a prerequisite for the existence of stem cells in normal tissues [6]. Stem cells are organized in a biologically distinct subset within the tumor mass known as a niche. Mechanical forces in the viscoelastic materials of tissues force stem cells to form niches [176]. Interestingly, in vitro studies in spheroid models have approached stem cell niche properties [177]. It is shown that the hypoxic core of the niche may present a selective advantage for stem cells, protecting them from oxidative stress. However, such conditions can also activate hypoxia-induced EMT pathways. Nevertheless, p-EMT seems to offer an advantage to CSC stemness compared to the “pure” epithelial or mesenchymal phenotype, probably due to the propensity of p-EMT cells to self-renewal and formation of hybrid multicellular clusters [171,178] (Figure 3).

In the quest for the cells that initiate metastasis, two models have been proposed, the hierarchic and the stochastic [179]. The hierarchic model posits that metastasis occurs when the pluripotential stem cells become tumorigenic. In contrast, the stochastic model posits that every tumor cell that undergoes EMT may acquire stem cell properties and become metastatic. Currently, the normal existence of stem cells in tissues and the EMT-favorable properties in the microenvironment of their niches has set new standards for detecting the cells that promote metastasis and for the targeted therapy against them [176]. However, the EMT-MET spectrum is dominated by several intermediate states of p-EMT cells that seem to play a key role in metastasis. Using mathematical models, Goetz et al. suggested that the higher number of “occult” intermediate states of p-EMT is associated with an accelerating effect on cancer metastasis [180].

Another recent point of interest in the EMT theory is the role of circulating tumor cells (CTC) [181]. These cells pass to the blood circulation in a partial EMT state, which provides a survival benefit. CTCs may remain dormant in the bone marrow until they find favorable conditions in a distant organ to create a metastatic niche. The level of detection of CTC in laryngeal carcinoma has been related to prognosis and response to therapy [182]. The ability to detect CTCs in the periphery may be a significant step in diagnosing metastatic carcinomas in the future, serving as a liquid biopsy. Cell-free tumor DNA (ctDNA), proteins, metabolites, exosomes, mRNA, and miRNAs detected in blood or saliva samples could emerge as additional promising biomarkers, acting as a liquid biopsy for laryngeal carcinoma [183].

The epithelial-to-mesenchymal transition already has promising clinical implications regarding prognosis and therapy. Altered expressions of key EMT molecules, such as Snail, Twist, MMP-9, vimentin, and E-cadherin, have proven their prognostic value in various carcinomas and are already established in clinical practice [184,185,186,187,188]. An ambitious therapeutic target could be the reversal of EMT. Clinical trials have been performed using flavonoids [189], the introduction of miRNA-200 [190], and inhibitors of TGF-β [191], EGFR [192], and ILK [193]. Knocking down Snail has been shown to enhance chemosensitivity [194]. In laryngeal carcinoma cell lines, successful attempts to reverse EMT have been made with the introduction of miRNAs [136,195,196,197,198], inhibitors of long-noncoding RNA [199,200], and flavonoids such as genistein, dihydroartemisinin, and brusatol [201,202,203]. Moreover, a higher expression of estrogen receptor-β has been found to correlate with lower EMT traits in laryngeal carcinoma. Consequently, the reversal of EMT in laryngeal carcinoma with estrogen receptor-β agonists has been suggested [204]. However, keeping in mind that the MET is a critical event in the homing of tumor cells in distant tissues, the optimal timing and effectiveness of therapeutic interventions on molecular pathways aiming at EMT reversal are still questionable [205].

In conclusion, EMT theory in the metastasis of epithelial carcinomas is based on sound scientific evidence. It is a theory that approaches the acquisition by tumor cells of migratory capacity and constitutes a holistic model that explains how an epithelial tumor develops all the necessary modifications to migrate, survive, and proliferate. EMT should be approached as a dynamic phenomenon of cell plasticity that partially characterizes specific cells within a particular time frame. The further elucidation of the EMT mechanisms, the multifunctional role of p-EMT cells, the focus on carcinoma stem cell niches, and the detection of circulating tumor cells in laryngeal carcinoma may highlight new useful molecular targets to assist diagnosis, prognosis, and therapy for this generally aggressive tumor.

## References

[1] Nocini R., Molteni G., Mattiuzzi C., Lippi G. (2020). Updates on larynx cancer epidemiology. Chin. J. Cancer Res..

[2] Suhail Y., Cain M.P., Vanaja K., Kurywchak P.A., Levchenko A., Kalluri R., Kshitiz (2019). Systems Biology of Cancer Metastasis. Cell Syst..

[3] Thiery J.P., Chopin D. (1999). Epithelial cell plasticity in development and tumor progression. Cancer Metastasis Rev..

[4] Lu W., Kang Y. (2019). Epithelial-Mesenchymal Plasticity in Cancer Progression and Metastasis. Dev. Cell.

[5] Marconi G.D., Fonticoli L., Rajan T.S., Pierdomenico S.D., Trubiani O., Pizzicannella J., Diomede F. (2021). Epithelial-Mesenchymal Transition (EMT): The Type-2 EMT in Wound Healing, Tissue Regeneration and Organ Fibrosis. Cells.

[6] Lambert A.W., Weinberg R.A. (2021). Linking EMT programmes to normal and neoplastic epithelial stem cells. Nat. Rev. Cancer.

[7] Garcia M.A., Nelson W.J., Chavez N. (2018). Cell-Cell Junctions Organize Structural and Signaling Networks. Cold Spring Harb. Perspect. Biol..

[8] Fan X., Jin S., Li Y., Khadaroo P.A., Dai Y., He L., Zhou D., Lin H. (2019). Genetic and Epigenetic Regulation of E-Cadherin Signaling in Human Hepatocellular Carcinoma. Cancer Manag. Res..

[9] Masterson J., O’Dea S. (2007). Posttranslational truncation of E-cadherin and significance for tumour progression. Cells Tissues Organs.

[10] Cai J., Culley M.K., Zhao Y., Zhao J. (2018). The role of ubiquitination and deubiquitination in the regulation of cell junctions. Protein Cell.

[11] Loh C.Y., Chai J.Y., Tang T.F., Wong W.F., Sethi G., Shanmugam M.K., Chong P.P., Looi C.Y. (2019). The E-Cadherin and N-Cadherin Switch in Epithelial-to-Mesenchymal Transition: Signaling, Therapeutic Implications, and Challenges. Cells.

[12] Cappellesso R., Marioni G., Crescenzi M., Giacomelli L., Guzzardo V., Mussato A., Staffieri A., Martini A., Blandamura S., Fassina A. (2015). The prognostic role of the epithelial-mesenchymal transition markers E-cadherin and Slug in laryngeal squamous cell carcinoma. Histopathology.

[13] Zhu G.J., Song P.P., Zhou H., Shen X.H., Wang J.G., Ma X.F., Gu Y.J., Liu D.D., Feng A.N., Qian X.Y. (2018). Role of epithelial-mesenchymal transition markers E-cadherin, N-cadherin, β-catenin and ZEB2 in laryngeal squamous cell carcinoma. Oncol. Lett..

[14] Nardi C.E., Dedivitis R.A., Camillo de Almeida R., de Matos L.L., Cernea C.R. (2018). The role of E-cadherin and β-catenin in laryngeal cancer. Oncotarget.

[15] Yu L., Li H.Z., Lu S.M., Tian J.J., Ma J.K., Wang H.B., Xu W. (2011). Downregulation of TWIST decreases migration and invasion of laryngeal carcinoma Hep-2 cells by regulating the E-cadherin, N-cadherin expression. J. Cancer Res. Clin. Oncol..

[16] Zhao X., Yu D., Yang J., Xue K., Liu Y., Jin C. (2017). Knockdown of Snail inhibits epithelial-mesenchymal transition of human laryngeal squamous cell carcinoma Hep-2 cells through the vitamin D receptor signaling pathway. Biochem. Cell Biol..

[17] Leggett S.E., Hruska A.M., Guo M., Wong L.Y. (2021). The epithelial-mesenchymal transition and the cytoskeleton in bioengineered systems. Cell Commun. Signal..

[18] Battaglia R.A., Delic S., Herrmann H., Snider N.T. (2018). Vimentin on the move: New developments in cell migration. F1000Research.

[19] Usman S., Waseem N.H., Nguyen T.K.N., Mohsin S., Jamal A., The M.T., Waseem A. (2021). Vimentin Is at the Heart of Epithelial Mesenchymal Transition (EMT) Mediated Metastasis. Cancers.

[20] Strouhalova K., Přechová M., Gandalovičová A., Brábek J., Gregor M., Rosel D. (2020). Vimentin Intermediate Filaments as Potential Target for Cancer Treatment. Cancers.

[21] Karamanou K., Franchi M., Vynios D., Brézillon S. (2020). Epithelial-to-mesenchymal transition and invadopodia markers in breast cancer: Lumican a key regulator. Semin. Cancer Biol..

[22] Manuelli V., Cahill F., Wylie H., Gillett C., Correa I., Heck S., Rimmer A., Haire A., Van Hemelrijck M., Rudman S. (2022). Invadopodia play a role in prostate cancer progression. BMC Cancer.

[23] van der Velden L.A., Schaafsma H.E., Manni J.J., Ruiter D.J., Ramaekers F.C., Kuijpers W. (1997). Cytokeratin and vimentin expression in normal epithelium and squamous cell carcinomas of the larynx. Eur. Arch. Otorhinolaryngol..

[24] Odero-Marah V., Hawsawi O., Henderson V., Sweeney J. (2018). Epithelial-Mesenchymal Transition (EMT) and Prostate Cancer. Adv. Exp. Med. Biol..

[25] Liu S., Zhou F., Shen Y., Zhang Y., Yin H., Zeng Y., Liu J., Yan Z., Liu X. (2016). Fluid shear stress induces epithelial-mesenchymal transition (EMT) in Hep-2 cells. Oncotarget.

[26] Ali A., Soares A.B., Eymael D., Magalhaes M. (2021). Expression of invadopodia markers can identify oral lesions with a high risk of malignant transformation. J. Pathol. Clin. Res..

[27] Castillo Ferrer C., Berthenet K., Ichim G., Paoli P., Giannoni E., Chiarugi P. (2021). Apoptosis-Fueling the oncogenic fire. FEBS J..

[28] Paoli P., Giannoni E., Chiarugi P. (2013). Anoikis molecular pathways and its role in cancer progression. Biochim. Biophys. Acta..

[29] Cao Z., Livas T., Kyprianou N. (2016). Anoikis and EMT: Lethal “Liaisons” during Cancer Progression. Crit. Rev. Oncog..

[30] Alanko J., Mai A., Jacquemet G., Schauer K., Kaukonen R., Saari M., Goud B., Ivaska J. (2015). Integrin endosomal signalling suppresses anoikis. Nat. Cell Biol..

[31] Kilinc A.N., Han S., Barrett L.A., Anandasivam N., Nelson C.M. (2021). Integrin-linked kinase tunes cell-cell and cell-matrix adhesions to regulate the switch between apoptosis and EMT downstream of TGFβ1. Mol. Biol. Cell.

[32] Tsirtsaki K., Gkretsi V. (2020). The focal adhesion protein Integrin-Linked Kinase (ILK) as an important player in breast cancer pathogenesis. Cell Adh. Migr..

[33] Lin J., Song T., Li C., Mao W. (2020). GSK-3β in DNA repair, apoptosis, and resistance of chemotherapy, radiotherapy of cancer. Biochim. Acta Mol. Cell Res..

[34] Gundamaraju R., Lu W., Paul M.K., Jha N.K., Gupta P.K., Ojha S., Chattopadhyay I., Rao P.V., Ghavami S. (2022). Autophagy and EMT in cancer and metastasis: Who controls whom?. Biochim. Biophys. Acta Mol. Basis Dis..

[35] Babaei G., Aziz S.G., Jaghi N.Z.Z. (2021). EMT, cancer stem cells and autophagy; The three main axes of metastasis. Biomed.Pharm..

[36] Gugnoni M., Sancisi V., Manzotti G., Gandolfi G., Ciarrocchi A. (2016). Autophagy and epithelial-mesenchymal transition: An intricate interplay in cancer. Cell Death Dis..

[37] Zhang Y., Sun X. (2020). Role of Focal Adhesion Kinase in Head and Neck Squamous Cell Carcinoma and Its Therapeutic Prospect. OncoTargets Ther..

[38] Chrysovergis A., Papanikolaou V., Tsiambas E., Kikidis D., Maragoudakis P., Ragos V., Kyrodimos E. (2019). Caspase complex in laryngeal squamous cell carcinoma. J. BUON.

[39] Han B.B., Li S., Tong M., Holpuch A.S., Spinney R., Wang D., Border M.B., Liu Z., Sarode S., Pei P. (2015). Fenretinide Perturbs Focal Adhesion Kinase in Premalignant and Malignant Human Oral Keratinocytes. Fenretinide’sChemopreventive Mechanisms Include ECM Interactions. Cancer Prev. Res..

[40] Xu Y.T., Chen R.Q., Lin G.B., Fang X.L., Yu S.J., Liang X.H., Zhang R. (2018). Defining the regulatory role of programmed cell death 4 in laryngeal squamous cell carcinoma. Biochem. Cell Biol..

[41] Larson C., Oronsky B., Carter C.A., Oronsky A., Knox S.J., Sher D., Reid T.R. (2020). TGF-beta: A master immune regulator. Expert Opin. Ther. Target.

[42] Batlle E., Massagué J. (2019). Transforming Growth Factor-β Signaling in Immunity and Cancer. Immunity.

[43] Liu M., Kuo F., Capistrano K.J., Kang D., Nixon B.G., Shi W., Chou C., Do M.H., Stamatiades E.G., Gao S. (2020). TGF-β suppresses type 2 immunity to cancer. Nature.

[44] Plaschka M., Benboubker V., Grimont M., Berthet J., Tonon L., Lopez J., Le-Bouar M., Balme B., Tondeur G., de la Fouchardiere A. (2022). ZEB1 transcription factor promotes immune escape in melanoma. J. Immunother. Cancer.

[45] Pang X., Tang Y.L., Liang X.H. (2018). Transforming growth factor-β signaling in head and neck squamous cell carcinoma: Insights into cellular responses. Oncol. Lett..

[46] Di Gioacchino M., Della Valle L., Allegra A., Pioggia G., Gangemi S. (2022). AllergoOncology: Role of immune cells and immune proteins. Clin. Transl. Allergy.

[47] Bhatt A.B., Patel S., Matossian M.D., Ucar D.A., Miele L., Burow M.E., Flaherty P.T., Cavanaugh J.E. (2021). Molecular Mechanisms of Epithelial to Mesenchymal Transition Regulated by ERK5 Signaling. Biomolecules.

[48] Liu J.F., Crépin M., Liu J.M., Barritault D., Ledoux D. (2002). FGF-2 and TPA induce matrix metalloproteinase-9 secretion in MCF-7 cells through PKC activation of the Ras/ERK pathway. Biochem. Biophys. Res. Commun..

[49] Zuo J.H., Zhu W., Li M.Y., Li X.H., Yi H., Zeng G.Q., Wan X.X., He Q.Y., Li J.H., Qu J.Q. (2011). Activation of EGFR promotes squamous carcinoma SCC10A cell migration and invasion via inducing EMT-like phenotype change and MMP-9-mediated degradation of E-cadherin. J. Cell. Biochem..

[50] Gonzalez-Avila G., Sommer B., García-Hernández A.A., Ramos C. (2020). Matrix Metalloproteinases’ Role in Tumor Microenvironment. Adv. Exp. Med. Biol..

[51] Turunen S.P., Tatti-Bugaeva O., Lehti K. (2017). Membrane-type matrix metalloproteases as diverse effectors of cancer progression. Biochim. Biophys. Acta Mol. Cell Res..

[52] Guo F., Liu J., Han X., Zhang X., Lin T., Wang Y., Bai J., Han J. (2019). FBXO22 Suppresses Metastasis in Human Renal Cell Carcinoma via Inhibiting MMP-9-Mediated Migration and Invasion and VEGF-Mediated Angiogenesis. Int. J. Biol. Sci..

[53] Niland S., Riscanevo A.X., Eble J.A. (2021). Matrix Metalloproteinases Shape the Tumor Microenvironment in Cancer Progression. Int. J. Mol. Sci..

[54] Heinz A. (2020). Elastases and elastokines: Elastin degradation and its significance in health and disease. Crit. Rev. Biochem. Mol. Biol..

[55] Christopoulos T.A., Papageorgakopoulou N., Ravazoula P., Mastronikolis N.S., Papadas T.A., Theocharis D.A., Vynios D.H. (2007). Expression of metalloproteinases and their tissue inhibitors in squamous cell laryngeal carcinoma. Oncol. Rep..

[56] Danilewicz M., Sikorska B., Wagrowska-Danilewicz M. (2003). Prognostic significance of the immunoexpression of matrix metalloproteinase MMP2 and its inhibitor TIMP2 in laryngeal cancer. Med. Sci. Monit..

[57] Wittekindt C., Jovanovic N., Guntinas-Lichius O. (2011). Expression of matrix metalloproteinase-9 (MMP-9) and blood vessel density in laryngeal squamous cell carcinomas. Acta Otolaryngol..

[58] Fu L.Q., Du W.L., Cai M.H., Yao J.Y., Zhao Y.Y., Mou X.Z. (2020). The roles of tumor-associated macrophages in tumor angiogenesis and metastasis. Cell Immunol..

[59] Gyftopoulos K., Vourda K., Sakellaropoulos G., Perimenis P., Athanasopoulos A., Papadaki E. (2011). The angiogenic switch for vascular endothelial growth factor-A and cyclooxygenase-2 in prostate carcinoma: Correlation with microvessel density, androgen receptor content and Gleason grade. Urol. Int..

[60] Zhang T., Liu L., Lai W., Zeng Y., Xu H., Lan Q., Su P., Chu Z. (2019). Interaction with tumor associated macrophages promotes PRL 3 induced invasion of colorectal cancer cells via MAPK pathway induced EMT and NF κB signaling induced angiogenesis. Oncol. Rep..

[61] Zhang J., Wang S., He Y., Yao B., Zhang Y. (2020). Regulation of matrix metalloproteinases 2 and 9 in corneal neovascularization. Chem. Biol. Drug Des..

[62] Stacker S.A., Achen M.G. (2018). Emerging Roles for VEGF-D in Human Disease. Biomolecules.

[63] Cao W., Xu C., Li X., Yang X. (2019). Twist1 promotes astrocytoma development by stimulating vasculogenic mimicry. Oncol. Lett..

[64] Luo Q., Wang J., Zhao W., Peng Z., Liu X., Li B., Zhang H., Shan B., Zhang C., Duan C. (2020). Vasculogenic mimicry in carcinogenesis and clinical applications. J.Hematol. Oncol..

[65] Franz L., Nicolè L., Frigo A.C., Ottaviano G., Gaudioso P., Saccardo T., Visconti F., Cappellesso R., Blandamura S., Fassina A. (2021). Epithelial-to-Mesenchymal Transition and Neoangiogenesis in Laryngeal Squamous Cell Carcinoma. Cancers.

[66] Lin P., Wang W., Sun B.C., Cai W.J., Li L., Lu H.H., Han C.R., Zhang J.M. (2012). Vasculogenic mimicry is a key prognostic factor for laryngeal squamous cell carcinoma: A new pattern of blood supply. Chin. Med. J..

[67] BolzoniVillaret A., Barbieri D., Peretti G., Schreiber A., Fisogni S., Lonardi S., Facchetti F., Nicolai P. (2013). Angiogenesis and lymphangiogenesis in early-stage laryngeal carcinoma: Prognostic implications. Head Neck.

[68] Oshimori N. (2020). Cancer stem cells and their niche in the progression of squamous cell carcinoma. Cancer Sci..

[69] Karatas O.F., Suer I., Yuceturk B., Yilmaz M., Hajiyev Y., Creighton C.J., Ittmann M., Ozen M. (2016). The role of miR-145 in stem cell characteristics of human laryngeal squamous cell carcinoma Hep-2 cells. Tumour Biol..

[70] Szafarowski T., Sierdziński J., Ludwig N., Głuszko A., Filipowska A., Szczepański M.J. (2020). Assessment of cancer stem cell marker expression in primary head and neck squamous cell carcinoma shows prognostic value for aldehyde dehydrogenase (ALDH1A1). Eur. J. Pharm..

[71] Elkashty O.A., Abu Elghanam G., Su X., Liu Y., Chauvin P.J., Tran S.D. (2020). Cancer stem cells enrichment with surface markers CD271 and CD44 in human head and neck squamous cell carcinomas. Carcinogenesis.

[72] Joshua B., Kaplan M.J., Doweck I., Pai R., Weissman I.L., Prince M.E., Ailles L.E. (2012). Frequency of cells expressing CD44, a head and neck cancer stem cell marker: Correlation with tumor aggressiveness. Head Neck.

[73] Dongre A., Weinberg R.A. (2019). New insights into the mechanisms of epithelial-mesenchymal transition and implications for cancer. Nat. Rev. Mol. Cell Biol..

[74] Catalano V., Turdo A., Di Franco S., Dieli F., Todaro M., Stassi G. (2013). Tumor and its microenvironment: A synergistic interplay. Semin. Cancer Biol..

[75] Bussard K.M., Mutkus L., Stumpf K., Gomez-Manzano C., Marini F.C. (2016). Tumor-associated stromal cells as key contributors to the tumor microenvironment. Breast Cancer Res..

[76] Caja L., Dituri F., Mancarella S., Caballero-Diaz D., Moustakas A., Giannelli G., Fabregat I. (2018). TGF-β and the Tissue Microenvironment: Relevance in Fibrosis and Cancer. Int. J. Mol. Sci..

[77] Asif P.J., Longobardi C., Hahne M., Medema J.P. (2021). The Role of Cancer-Associated Fibroblasts in Cancer Invasion and Metastasis. Cancers.

[78] Akhtar M., Haider A., Rashid S., Al-Nabet A.D.M.H. (2019). Paget’s “Seed and Soil” Theory of Cancer Metastasis: An Idea Whose Time has Come. Adv. Anat. Pathol..

[79] Custódio M., Biddle A., Tavassoli M. (2020). Portrait of a CAF: The story of cancer-associated fibroblasts in head and neck cancer. Oral Oncol..

[80] OrgenCalli A., Dere Y., Sari A., Dirilenoglu F., Onur I., İmre K. (2019). Evaluation of Stromal Myofibroblasts in Laryngeal Dysplasia and Invasive Squamous Cell Carcinoma. Indian J. Otolaryngol. Head Neck Surg..

[81] Huang Q., Yang J., Zheng J., Hsueh C., Guo Y., Zhou L. (2018). Characterization of selective exosomal microRNA expression profile derived from laryngeal squamous cell carcinoma detected by next generation sequencing. Oncol. Rep..

[82] Wu C., Wang M., Huang Q., Guo Y., Gong H., Hu C., Zhou L. (2022). Aberrant expression profiles and bioinformatic analysis of CAF-derived exosomal miRNAs from three moderately differentiated supraglottic LSCC patients. J. Clin. Lab. Anal..

[83] Zhao Q., Zheng X., Guo H., Xue X., Zhang Y., Niu M., Cui J., Liu H., Luo H., Yang D. (2020). Serum Exosomal miR-941 as a promising Oncogenic Biomarker for Laryngeal Squamous Cell Carcinoma. J. Cancer.

[84] Gonzalez D.M., Medici D. (2014). Signaling mechanisms of the epithelial-mesenchymal transition. Sci. Signal..

[85] Xu J., Lamouille S., Derynck R. (2009). TGF-beta-induced epithelial to mesenchymal transition. Cell Res..

[86] Hao Y., Baker D., Ten Dijke P. (2019). TGF-β-Mediated Epithelial-Mesenchymal Transition and Cancer Metastasis. Int. J. Mol. Sci..

[87] Sader F., Denis J.F., Laref H., Roy S. (2019). Epithelial to mesenchymal transition is mediated by both TGF-β canonical and non-canonical signaling during axolotl limb regeneration. Sci. Rep..

[88] Hagedorn H., Sauer U., Schleicher E., Nerlich A. (1999). Expression of TGF-beta 1 protein and mRNA and the effect on the tissue remodeling in laryngeal carcinomas. Anticancer Res..

[89] Zheng L., Guan Z., Xue M. (2022). TGF-β Signaling Pathway-Based Model to Predict the Subtype and Prognosis of Head and Neck Squamous Cell Carcinoma. Front. Genet..

[90] Franchi A., Gallo O., Sardi I., Santucci M. (2001). Downregulation of transforming growth factor beta type II receptor in laryngeal carcinogenesis. J. Clin. Pathol..

[91] Zhang Y., Wang X. (2020). Targeting the Wnt/β-catenin signaling pathway in cancer. J. Hematol. Oncol..

[92] Castellone M.D., Laukkanen M.O. (2017). TGF-beta1, WNT, and SHH signaling in tumor progression and in fibrotic diseases. Front. Biosci..

[93] Patel S., Alam A., Pant R., Chattopadhyay S. (2019). Wnt Signaling and Its Significance Within the Tumor Microenvironment: Novel Therapeutic Insights. Front. Immunol..

[94] Sun S., Gong C., Yuan K. (2019). LncRNA UCA1 promotes cell proliferation, invasion and migration of laryngeal squamous cell carcinoma cells by activating Wnt/β-catenin signaling pathway. Exp. Ther. Med..

[95] Zhang F., Mao D., He Z., Li W., Zhang X., Li L. (2021). SLCO4A1-AS1 regulates laryngeal squamous cell carcinoma cell phenotypes via the Wnt pathway. Oral Dis..

[96] Cui X., Fang N., Cui Y., Xiao D., Wang X. (2019). Long non-coding RNA NEF inhibits proliferation and promotes apoptosis of laryngeal squamous cell carcinoma cells by inhibiting Wnt/β-catenin signaling. Oncol. Lett..

[97] Shi J., Wang J., Cheng H., Liu S., Hao X., Lan L., Wu G., Liu M., Zhao Y. (2021). FOXP4 promotes laryngeal squamous cell carcinoma progression through directly targeting LEF 1. Mol. Med. Rep..

[98] Tang X., Sun Y., Wan G., Sun J., Sun J., Pan C. (2019). Knockdown of YAP inhibits growth in Hep-2 laryngeal cancer cells via epithelial-mesenchymal transition and the Wnt/β-catenin pathway. BMC Cancer.

[99] Psyrri A., Kotoula V., Fountzilas E., Alexopoulou Z., Bobos M., Televantou D., Karayannopoulou G., Krikelis D., Markou K., Karasmanis I. (2014). Prognostic significance of the Wnt pathway in squamous cell laryngeal cancer. Oral Oncol..

[100] Ghosh S., Marrocco I., Yarden Y. (2020). Roles for receptor tyrosine kinases in tumor progression and implications for cancer treatment. Adv. Cancer Res..

[101] Tripathi K., Garg M. (2018). Mechanistic regulation of epithelial-to-mesenchymal transition through RAS signaling pathway and therapeutic implications in human cancer. J. Cell Commun. Signal..

[102] Jansen S., Gosens R., Wieland T., Schmidt M. (2018). Paving the Rho in cancer metastasis: Rho GTPases and beyond. Pharmacol. Ther..

[103] Guarino M. (2010). Src signaling in cancer invasion. J. Cell. Physiol..

[104] Liu X., Yun F., Shi L., Li Z.H., Luo N.R., Jia Y.F. (2015). Roles of Signaling Pathways in the Epithelial-Mesenchymal Transition in Cancer. Asian Pac. J. Cancer Prev..

[105] Song Y., Dong Y.D., Bai W.L., Ma X.L. (2014). Silencing of Src by siRNA inhibits laryngeal carcinoma growth through the Src/PI-3 K/Akt pathway in vitro and in vivo. Tumour Biol..

[106] Dong L.B., Li G.Q., Tian Z.H., Wang Z.M., Xu K. (2013). Expressions of Src homology 2 domain-containing phosphatase and its clinical significance in laryngeal carcinoma. Genet. Mol. Res..

[107] Porcheri C., Meisel C.T., Mitsiadis T. (2019). Multifactorial Contribution of Notch Signaling in Head and Neck Squamous Cell Carcinoma. Int. J. Mol. Sci..

[108] Zou Y., Fang F., Ding Y.J., Dai M.Y., Yi X., Chen C., Tao Z.Z., Chen S.M. (2016). Notch 2 signaling contributes to cell growth, anti-apoptosis and metastasis in laryngeal squamous cell carcinoma. Mol. Med. Rep..

[109] Dai M.Y., Fang F., Zou Y., Yi X., Ding Y.J., Chen C., Tao Z.Z., Chen S.M. (2015). Downregulation of Notch1 induces apoptosis and inhibits cell proliferation and metastasis in laryngeal squamous cell carcinoma. Oncol. Rep..

[110] Papanikolaou V., Chrysovergis A., Mastronikolis S., Tsiambas E., Ragos V., Peschos D., Spyropoulou D., Pantos P., Niotis A., Mastronikolis N. (2021). Impact of K-Ras Over-expression in Laryngeal Squamous Cell Carcinoma. In Vivo.

[111] Ren J., Zhu D., Liu M., Sun Y., Tian L. (2010). Downregulation of miR-21 modulates Ras expression to promote apoptosis and suppress invasion of Laryngeal squamous cell carcinoma. Eur. J. Cancer.

[112] Sun Z., Guo S.S., Fässler R. (2016). Integrin-mediated mechanotransduction. J. Cell Biol..

[113] Legerstee K., Houtsmuller A.B. (2021). A Layered View on Focal Adhesions. Biology.

[114] Shams H., Hoffman B.D., Mofrad M.R.K. (2018). The “Stressful” Life of Cell Adhesion Molecules: On the Mechanosensitivity of Integrin Adhesome. J. Biomech. Eng..

[115] Li M., Wang Y., Li M., Wu X., Setrerrahmane S., Xu H. (2021). Integrins as attractive targets for cancer therapeutics. Acta Pharm. Sin. B.

[116] Li F., Liu Y., Kan X., Li Y., Liu M., Lu J.G. (2013). Elevated expression of integrin αv and β5 subunit in laryngeal squamous-cell carcinoma associated with lymphatic metastasis and angiogenesis. Pathol. Res. Pract..

[117] Dong X., Luo Z., Liu T., Chai J., Ke Q., Shen L. (2018). Identification of Integrin β1 as a Novel PAG1-Interacting Protein Involved in the Inherent Radioresistance of Human Laryngeal Carcinoma. J. Cancer.

[118] Lu J.G., Li Y., Li L., Kan X. (2011). Overexpression of osteopontin and integrin αv in laryngeal and hypopharyngeal carcinomas associated with differentiation and metastasis. J. Cancer Res. Clin. Oncol..

[119] Lu J.G., Sun Y.N., Wang C., Jin D.J., Liu M. (2009). Role of the alpha v-integrin subunit in cell proliferation, apoptosis and tumor metastasis of laryngeal and hypopharyngeal squamous cell carcinomas: A clinical and in vitro investigation. Eur. Arch. Otorhinolaryngol..

[120] Vitolo D., Ciocci L., Ferrauti P., Cicerone E., Gallo A., De Vincentiis M., Baroni C.D. (2000). alpha5 integrin distribution and TGFbeta1 gene expression in supraglottic carcinoma: Their role in neoplastic local invasion and metastasis. Head Neck.

[121] Pan G., Liu Y., Shang L., Zhou F., Yang S. (2021). EMT-associated microRNAs and their roles in cancer stemness and drug resistance. Cancer Commun..

[122] MusaviShenas M.H., Eghbal-Fard S., Mehrisofiani V., Abd Yazdani N., Rahbar Farzam O., Marofi F., Yousefi M. (2019). MicroRNAs and signaling networks involved in epithelial-mesenchymal transition. J. Cell Physiol..

[123] Lin S., Gregory R.I. (2015). MicroRNA biogenesis pathways in cancer. Nat. Rev. Cancer.

[124] Hill L., Browne G., Tulchinsky E. (2013). ZEB/miR-200 feedback loop: At the crossroads of signal transduction in cancer. Int. J. Cancer.

[125] Gregory P.A., Bracken C.P., Smith E., Bert A.G., Wright J.A., Roslan S., Morris M., Wyatt L., Farshid G., Lim Y.-Y. (2011). An autocrine TGF-beta/ZEB/miR-200 signaling network regulates establishment and maintenance of epithelial-mesenchymal transition. Mol. Biol. Cell.

[126] Fang C.Y., Yu C.C., Liao Y.W., Hsieh P.L., Ohiro Y., Chu P.M., Huang Y.C., Yu C.H., Tsai L.L. (2020). miR-10b regulated by Twist maintains myofibroblasts activities in oral submucous fibrosis. J. Formos. Med. Assoc..

[127] Bourguignon L.Y., Wong G., Earle C., Krueger K., Spevak C.C. (2010). Hyaluronan-CD44 interaction promotes c-Src-mediated twist signaling, microRNA-10b expression, and RhoA/RhoC upregulation, leading to Rho-kinase-associated cytoskeleton activation and breast tumor cell invasion. J. Biol. Chem..

[128] Wu C., Peng S., Sun W., Luo M., Su B., Liu D., Hu G. (2018). Association of E-cadherin methylation with risk of nasopharyngeal cancer: A meta-analysis. Head Neck.

[129] Yang C.X., Sedhom W., Song J., Lu S.L. (2019). The Role of MicroRNAs in Recurrence and Metastasis of Head and Neck Squamous Cell Carcinoma. Cancers.

[130] Chen L., Sun D.Z., Fu Y.G., Yang P.Z., Lv H.Q., Gao Y., Zhang X.Y. (2020). Upregulation of microRNA-141 suppresses epithelial-mesenchymal transition and lymph node metastasis in laryngeal cancer through HOXC6-dependent TGF-β signaling pathway. Cell Signal..

[131] Yang B., Zang J., Yuan W., Jiang X., Zhang F. (2021). The miR-136-5p/ROCK1 axis suppresses invasion and migration, and enhances cisplatin sensitivity in head and neck cancer cells. Exp. Ther. Med..

[132] Wang H., Qian J., Xia X., Ye B. (2020). Long non-coding RNA OIP5-AS1 serves as an oncogene in laryngeal squamous cell carcinoma by regulating miR-204-5p/ZEB1 axis. Naunyn-Schmiedebergs Arch. Pharm..

[133] Zhang F., Cao H. (2019). MicroRNA 143 3p suppresses cell growth and invasion in laryngeal squamous cell carcinoma via targeting the k Ras/Raf/MEK/ERK signaling pathway. Int. J. Oncol..

[134] Gao W., Zhang C., Li W., Li H., Sang J., Zhao Q., Bo Y., Luo H., Zheng X., Lu Y. (2019). Promoter Methylation-Regulated miR-145-5p Inhibits Laryngeal Squamous Cell Carcinoma Progression by Targeting FSCN1. Mol. Ther..

[135] Zhang S.Y., Lu Z.M., Lin Y.F., Chen L.S., Luo X.N., Song X.H., Chen S.H., Wu Y.L. (2016). miR-144-3p, a tumor suppressive microRNA targeting ETS-1 in laryngeal squamous cell carcinoma. Oncotarget.

[136] Zhou M., Wang Y., Zhang C., Qi M., Yao M., Sun L., Xu X. (2021). MicroRNA-195-5p suppresses the proliferation, migration, invasion and epithelial-mesenchymal transition of laryngeal cancer cells in vitro by targeting E2F3. Exp. Ther. Med..

[137] Li Y., Tao C., Dai L., Cui C., Chen C., Wu H., Wei Q., Zhou X. (2019). MicroRNA-625 inhibits cell invasion and epithelial-mesenchymal transition by targeting SOX4 in laryngeal squamous cell carcinoma. Biosci. Rep..

[138] Zhu M., Zhang C., Chen D., Chen S., Zheng H. (2019). MicroRNA-98-HMGA2-POSTN signal pathway reverses epithelial-to-mesenchymal transition in laryngeal squamous cell carcinoma. Biomed. Pharm..

[139] Tian L., Li M., Ge J., Guo Y., Sun Y., Liu M., Xiao H. (2014). MiR-203 is downregulated in laryngeal squamous cell carcinoma and can suppress proliferation and induce apoptosis of tumours. Tumour Biol..

[140] Zhang L., Sun J., Wang B., Ren J.C., Su W., Zhang T. (2015). MicroRNA-10b Triggers the Epithelial-Mesenchymal Transition (EMT) of Laryngeal Carcinoma Hep-2 Cells by Directly Targeting the E-cadherin. Appl. Biochem. Biotechnol..

[141] Wang B., Lv K., Chen W., Zhao J., Luo J., Wu J., Li Z., Qin H., Wong T.S., Yang W. (2016). miR-375 and miR-205 Regulate the Invasion and Migration of Laryngeal Squamous Cell Carcinoma Synergistically via AKT-Mediated EMT. Biomed. Res. Int..

[142] Xu Y. (2022). MiRNA-21-5p Accelerates EMT and Inhibits Apoptosis of Laryngeal Carcinoma via Inhibiting KLF6 Expression. Biochem. Genet..

[143] Lu Y., Gao W., Zhang C., Wen S., Huangfu H., Kang J., Wang B. (2015). Hsa-miR-301a-3p Acts as an Oncogene in Laryngeal Squamous Cell Carcinoma via Target Regulation of Smad4. J. Cancer.

[144] Fuxe J., Karlsson M.C. (2012). TGF-β-induced epithelial-mesenchymal transition: A link between cancer and inflammation. Semin. Cancer Biol..

[145] Neil J.R., Johnson K.M., Nemenoff R.A., Schiemann W.P. (2008). Cox-2 inactivates Smad signaling and enhances EMT stimulated by TGF-beta through a PGE2-dependent mechanisms. Carcinogenesis.

[146] Tan J.J., Wang L., Mo T.T., Wang J., Wang M.G., Li X.P. (2019). Pepsin promotes IL-8 signaling-induced epithelial-mesenchymal transition in laryngeal carcinoma. Cancer Cell Int..

[147] Chattopadhyay I., Ambati R., Gundamaraju R. (2021). Exploring the Crosstalk between Inflammation and Epithelial-Mesenchymal Transition in Cancer. Mediat. Inflamm..

[148] Pezzuto A., Carico E. (2018). Role of HIF-1 in Cancer Progression: Novel Insights. A Review. Curr. Mol. Med..

[149] Yang M.H., Wu K.J. (2008). TWIST activation by hypoxia inducible factor-1 (HIF-1): Implications in metastasis and development. Cell Cycle.

[150] Zuo J., Wen J., Lei M., Wen M., Li S., Lv X., Luo Z., Wen G. (2016). Hypoxia promotes the invasion and metastasis of laryngeal cancer cells via EMT. Med. Oncol..

[151] Bao Y.Y., Zhou S.H., Lu Z.J., Fan J., Huang Y.P. (2015). Inhibiting GLUT-1 expression and PI3K/Akt signaling using apigenin improves the radiosensitivity of laryngeal carcinoma in vivo. Oncol. Rep..

[152] Jiang T., Zhou M.L., Fan J. (2018). Inhibition of GLUT-1 expression and the PI3K/Akt pathway to enhance the chemosensitivity of laryngeal carcinoma cells in vitro. OncoTargets Ther..

[153] Wu X.H., Lu Y.F., Hu X.D., Mao J.Y., Ji X.X., Yao H.T., Zhou S.H. (2010). Expression of hypoxia inducible factor-1α and its significance in laryngeal carcinoma. J. Int. Med. Res..

[154] Li D.W., Zhou L., Jin B., Xie J., Dong P. (2013). Expression and significance of hypoxia-inducible factor-1α and survivin in laryngeal carcinoma tissue and cells. Otolaryngol. Head Neck Surg..

[155] Zhao Z., Zhu X., Cui K., Mancuso J., Federley R., Fischer K., Teng G., Mittal V., Gao D., Zhao H. (2016). In Vivo Visualization and Characterization of Epithelial-Mesenchymal Transition in Breast Tumors. Cancer Res..

[156] Janani G., Pillai M.M., Selvakumar R., Bhattacharyya A., Sabarinath C. (2017). An in vitro 3D model using collagen coated gelatin nanofibers for studying breast cancer metastasis. Biofabrication.

[157] Jolly M.K., Boareto M., Huang B., Jia D., Lu M., Ben-Jacob E., Onuchic J.N., Levine H. (2015). Implications of the Hybrid Epithelial/Mesenchymal Phenotype in Metastasis. Front. Oncol..

[158] Bakir B., Chiarella A.M., Pitarresi J.R., Rustgi A.K. (2020). EMT, MET, Plasticity, and Tumor Metastasis. Trends Cell Biol..

[159] Barasch J. (2001). Genes and proteins involved in mesenchymal to epithelial transition. Curr. Opin. Nephrol. Hypertens..

[160] Sinha D., Saha P., Samanta A., Bishayee A. (2020). Emerging Concepts of Hybrid Epithelial-to-Mesenchymal Transition in Cancer Progression. Biomolecules.

[161] Saitoh M. (2018). Involvement of partial EMT in cancer progression. J. Biochem..

[162] Muralidharan S., Sahoo S., Saha A., Chandran S., Majumdar S.S., Mandal S., Levine H., Jolly M.K. (2022). Quantifying the Patterns of Metabolic Plasticity and Heterogeneity along the Epithelial-Hybrid-Mesenchymal Spectrum in Cancer. Biomolecules.

[163] Brabletz S., Schuhwerk H., Brabletz T., Stemmler M.P. (2021). Dynamic EMT: A multi-tool for tumor progression. EMBO J..

[164] Kisoda S., Mouri Y., Kitamura N., Yamamoto T., Miyoshi K., Kudo Y. (2022). The role of partial-EMT in the progression of head and neck squamous cell carcinoma. J. Oral Biosci..

[165] Pal A., Barrett T.F., Paolini R., Parikh A., Puram S.V. (2021). Partial EMT in head and neck cancer biology: A spectrum instead of a switch. Oncogene.

[166] Kisoda S., Shao W., Fujiwara N., Mouri Y., Tsunematsu T., Jin S., Arakaki R., Ishimaru N., Kudo Y. (2020). Prognostic value of partial EMT-related genes in head and neck squamous cell carcinoma by a bioinformatic analysis. Oral Dis..

[167] Fan L., Wang J., Deng P., Wang Y., Zhang A., Yang M., Zeng G. (2022). Foxhead box D1 promotes the partial epithelial-to-mesenchymal transition of laryngeal squamous cell carcinoma cells via transcriptionally activating the expression of zinc finger protein 532. Bioengineered.

[168] Liao C., Wang Q., An J., Long Q., Wang H., Xiang M., Xiang M., Zhao Y., Liu Y., Liu J. (2021). Partial EMT in Squamous Cell Carcinoma: A Snapshot. Int. J. Biol. Sci..

[169] Pastushenko I., Mauri F., Song Y., de Cock F., Meeusen B., Swedlund B., Impens F., Van Haver D., Opitz M., Thery M. (2021). Fat1 deletion promotes hybrid EMT state, tumour stemness and metastasis. Nature.

[170] Majidpoor J., Mortezaee K. (2021). Steps in metastasis: An updated review. Med. Oncol..

[171] Aggarwal V., Montoya C.A., Donnenberg V.S., Sant S. (2021). Interplay between tumor microenvironment and partial EMT as the driver of tumor progression. iScience.

[172] Labernadie A., Kato T., Brugués A., Serra-Picamal X., Derzsi S., Arwert E., Weston A., González-Tarragó V., Elosegui-Artola A., Albertazzi L. (2017). A mechanically active heterotypic E-cadherin/N-cadherin adhesion enables fibroblasts to drive cancer cell invasion. Nat. Cell Biol..

[173] Aiello N.M., Maddipati R., Norgard R.J., Balli D., Li J., Yuan S., Yamazoe T., Black T., Sahmoud A., Furth E.E. (2018). EMT subtype influences epithelial plasticity and mode of cell migration. Dev. Cell.

[174] Yang C., Cao M., Liu Y., He Y., Du Y., Zhang G., Gao F. (2019). Inducible formation of leadercells driven by CD44 switching gives rise to collective invasion and metastases in luminal breast carcinomas. Oncogene.

[175] Christofori G. (2006). New signals from the invasive front. Nature.

[176] Vining K.H., Mooney D.J. (2017). Mechanical forces direct stem cell behaviour in development and regeneration. Nat. Rev. Mol. Cell Biol..

[177] Forte E., Chimenti I., Rosa P., Angelini F., Pagano F., Calogero A., Giacomello A., Messina E. (2017). EMT/MET at the Crossroad of Stemness, Regeneration and Oncogenesis: The Ying-Yang Equilibrium Recapitulated in Cell Spheroids. Cancers.

[178] Jolly M.K., Somarelli J.A., Sheth M., Biddle A., Tripathi S.C., Armstrong A.J., Hanash S.M., Bapat S.A., Ranagarajan A., Levine H. (2019). Hybrid epithelial/mesenchymal phenotypes promote metastasis and therapy resistance across carcinomas. Pharmacol. Ther..

[179] Plaks V., Kong N., Werb Z. (2015). The cancer stem cell niche: How essential is the niche in regulating stemness of tumor cells?. Cell Stem Cell.

[180] Goetz H., Melendez-Alvarez J.R., Chen L., Tian X.J. (2020). A plausible accelerating function of intermediate states in cancer metastasis. PLoS Comput. Biol..

[181] Lowes L.E., Allan A.L. (2018). Circulating Tumor Cells and Implications of the Epithelial-to-Mesenchymal Transition. Adv. Clin. Chem..

[182] Rizzo M.I., Ralli M., Nicolazzo C., Gradilone A., Carletti R., Di Gioia C., De Vincentiis M., Greco A. (2020). Detection of circulating tumor cells in patients with laryngeal cancer using ScreenCell: Comparative pre- and post-operative analysis and association with prognosis. Oncol. Lett..

[183] Kong L., Birkeland A.C. (2021). Liquid Biopsies in Head and Neck Cancer: Current State and Future Challenges. Cancers.

[184] Jung A.R., Jung C.H., Noh J.K., Lee Y.C., Eun Y.G. (2020). Epithelial-mesenchymal transition gene signature is associated with prognosis and tumor microenvironment in head and neck squamous cell carcinoma. Sci. Rep..

[185] Zhang P., Hu P., Shen H., Yu J., Liu Q., Du J. (2014). Prognostic role of Twist or Snail in various carcinomas: A systematic review and meta-analysis. Eur. J. Clin. Investig..

[186] Gong L., Wu D., Zou J., Chen J., Chen L., Chen Y., Ni C., Yuan H. (2016). Prognostic impact of serum and tissue MMP-9 in non-small cell lung cancer: A systematic review and meta-analysis. Oncotarget.

[187] Chen Z., Fang Z., Ma J. (2021). Regulatory mechanisms and clinical significance of vimentin in breast cancer. Biomed. Pharm..

[188] Zhang X., Zhang Z., Chen S., Jiang J., Qi R., Mi X., Zhang X., Xi Y., Zheng H., Hua B. (2020). Prognostic significance of E-cadherin expression in prostatic carcinoma: A protocol for systematic review and meta-analysis. Medicine.

[189] Lv F., Du Q., Li L., Xi X., Liu Q., Li W., Liu S. (2021). Eriodictyol inhibits glioblastoma migration and invasion by reversing EMT via downregulation of the P38 MAPK/GSK-3β/ZEB1 pathway. Eur. J. Pharm..

[190] Lamouille S., Subramanyam D., Blelloch R., Derynck R. (2013). Regulation of epithelial-mesenchymal and mesenchymal-epithelial transitions by microRNAs. Curr. Opin. Cell Biol..

[191] Chen B., Zhou S., Zhan Y., Ke J., Wang K., Liang Q., Hou Y., Zhu P., Ao W., Wei X. (2019). Dioscin Inhibits the Invasion and Migration of Hepatocellular Carcinoma HepG2 Cells by Reversing TGF-β1-Induced Epithelial-Mesenchymal Transition. Molecules.

[192] Lin C., Ren Z., Yang X., Yang R., Chen Y., Liu Z., Dai Z., Zhang Y., He Y., Zhang C. (2020). Nerve growth factor (NGF)-TrkA axis in head and neck squamous cell carcinoma triggers EMT and confers resistance to the EGFR inhibitor erlotinib. Cancer Lett..

[193] Zhang Y., Huang W. (2018). Transforming Growth Factor β1 (TGF-β1)-Stimulated Integrin-Linked Kinase (ILK) Regulates Migration and Epithelial-Mesenchymal Transition (EMT) of Human Lens Epithelial Cells via Nuclear Factor κB (NF-κB). Med. Sci.Monit..

[194] Kaşıkcı E., Aydemir E., Bayrak Ö.F., Şahin F. (2020). Inhibition of Migration, Invasion and Drug Resistance of Pancreatic Adenocarcinoma Cells-Role of Snail, Slug and Twist and Small Molecule Inhibitors. Onco Targets Ther..

[195] Wu Y., Dai F., Zhang Y., Zheng X., Li L., Zhang Y., Cao J., Gao W. (2021). miR-1207-5p suppresses laryngeal squamous cell carcinoma progression by downregulating SKA3 and inhibiting epithelial-mesenchymal transition. Mol. Ther. Oncolytics.

[196] Lu B., Yu Y., Xing X.L., Liu R.Y. (2022). miR-183/TMSB4Y, a new potential signaling axis, involving in the progression of laryngeal cancer via modulating cell adhesion. J. Recept. Signal Transduct. Res..

[197] Li D.J., Wang X., Yin W.H., Niu K., Zhu W., Fang N. (2020). MiR-199a-5p suppresses proliferation and invasion of human laryngeal cancer cells. Eur. Rev. Med. Pharm. Sci..

[198] Li X., Wu P., Tang Y., Fan Y., Liu Y., Fang X., Wang W., Zhao S. (2020). Down-Regulation of MiR-181c-5p Promotes Epithelial-to-Mesenchymal Transition in Laryngeal Squamous Cell Carcinoma via Targeting SERPINE1. Front. Oncol..

[199] Wu X., Tan Y., Tang X. (2022). Long Noncoding RNA MALAT1 Promotes Laryngocarcinoma Development by Targeting miR-708-5p/BRD4 Axis to Regulate YAP1-Mediated Epithelial-Mesenchymal Transition. Biomed. Res. Int..

[200] Yang C., Cao H., Yang J.-W., Wang J.-T., Yu M.-M., Wang B.-S. (2022). The ETS1-LINC00278 negative feedback loop plays a role in COL4A1/COL4A2 regulation in laryngeal squamous cell carcinoma. Neoplasma.

[201] Du R., Liu Z., Hou X., Fu G., An N., Wang L. (2016). Trichostatin A potentiates genistein-induced apoptosis and reverses EMT in HEp2 cells. Mol. Med. Rep..

[202] Sun Y., Lu X., Li H., Li X. (2021). Dihydroartemisinin inhibits IL-6-induced epithelial-mesenchymal transition in laryngeal squamous cell carcinoma via the miR-130b-3p/STAT3/β-catenin signaling pathway. J. Int. Med. Res..

[203] Zhou J., Hou J., Wang J., Wang J., Gao J., Bai Y. (2021). Brusatol inhibits laryngeal cancer cell proliferation and metastasis via abrogating JAK2/STAT3 signaling mediated epithelial-mesenchymal transition. Life Sci..

[204] Goulioumis A.K., Fuxe J., Varakis J., Repanti M., Goumas P., Papadaki H. (2009). Estrogen receptor-beta expression in human laryngeal carcinoma: Correlation with the expression of epithelial-mesenchymal transition specific biomarkers. Oncol. Rep..

[205] Jolly M.K., Ware K.E., Gilja S., Somarelli J.A., Levine H. (2017). EMT and MET: Necessary or permissive for metastasis?. Mol. Oncol..

